# Canine Fecal Microbiome Dataset: Ultra-deep Multi-platform Sequencing Across Extraction and Library Protocols

**DOI:** 10.1038/s41597-026-07594-5

**Published:** 2026-06-09

**Authors:** Balázs Kakuk, Ákos Dörmő, Ahmed Taifi, Tamás Járay, Gábor Kurucsai, Gábor Gulyás, István Prazsák, Zsolt Boldogkői, Dóra Tombácz

**Affiliations:** 1 MTA-SZTE Lendület GeMiNI Research Group, University of Szeged, Somogyi st. 4., 6720 Szeged, Hungary; 2 Department of Medical Biology, Albert Szent-Györgyi Medical School, University of Szeged, Somogyi st. 4., 6720 Szeged, Hungary; 3 Kurucsai Medical Care Center, Berényi st. 23, 8000 Székesfehérvár, Hungary

**Keywords:** Microbiome, Metagenomics, Bacteriophages, DNA sequencing

## Abstract

The canine gut microbiome is an important model for microbiome research, yet methodological variation in DNA isolation, library preparation, and sequencing complicates cross-study comparisons. Here we present a three-component dataset to evaluate methodological effects. First, an ultra-deep sequencing dataset was generated from a single dog fecal sample using both short- (Illumina NovaSeq) and long-read (Oxford Nanopore MinION) platforms. Second, fecal samples from eight co-housed dogs were collected over one year to compare two DNA extraction workflows across 40 samples. Third, three full-length 16S rRNA primer sets were evaluated using synthetic microbial community standards and human and canine fecal samples, all sequenced on the MinION platform. The dataset comprises 75.2 GB of raw sequencing data and quality control and taxonomic classification outputs. The single-sample multi-platform dataset contributes 9.19 GB, the longitudinal cohort 43.45 GB, and the primer comparison dataset 22.61 GB across two accessions. Together, these data provide a multi-platform resource for evaluating extraction, sequencing, and primer-associated methodological effects in canine fecal microbiome profiling.

## Background & Summary

Investigating the microbiome is critical for unraveling how microbial communities influence both health and disease. However, it faces persistent technical challenges arising from the lack of universal standards in DNA isolation, library preparation, sequencing, and bioinformatics workflows - all of which can introduce biases that affect reproducibility and comparability across studies^1^. DNA extraction is a particularly influential step; different kits vary in their efficiency at lysing microbial cells or retaining certain taxa, potentially altering observed community profiles^2,3^. Researchers must then choose between targeted 16S rRNA gene amplicon sequencing and whole-genome shotgun (WGS) sequencing, each of which varies in cost, resolution, and coverage^4,5^. Sequencing platforms further complicate matters: while short-read sequencing (SRS) technologies (e.g., Illumina) are cost-effective and high-throughput, they struggle with highly repetitive or complex regions; conversely, long-read sequencing (LRS) platforms [Oxford Nanopore Technologies (ONT), Pacific Biosciences (PacBio)] enable more complete assemblies but require higher per-base costs and specialized protocols^6–10^. One of the most critical sources of bias in 16S rRNA gene studies is primer design. Conventional primers can fail to amplify essential taxa if mismatches occur in the conserved regions they target. Two recent studies underscore this issue: Matsuo *et al*.^11^ demonstrated that the “27 F” primer in the ONT 16S Barcoding Kit underrepresented *Bifidobacterium* and proposed degenerate primers to address this shortfall^11^. Waechter *et al*.^12^ later validated these degenerate primers in a large human fecal dataset, reporting improved capture of microbial diversity and reduced taxonomic biases^12^. Together, these findings suggest that even minimal primer modifications can significantly enhance community profiling, particularly at the species level.

While most microbiome research focuses on humans, the domestic dog (*Canis lupus familiaris*) represents an important comparative model with relevance to both veterinary and human health^13,14^. Dogs share close living environments, diets, and lifestyles with humans, all of which influence microbiome composition. In addition, dogs exhibit anatomical, physiological, and spontaneous disease similarities to humans that contribute to their translational value. Whole-genome metagenomic studies indicate that the canine gut microbiome is more similar to that of humans than to those of commonly used laboratory animals such as pigs or mice, both in gene content and in responses to dietary interventions^15^. As such, investigating the canine gut microbiome can improve understanding of host–microbiome interactions while providing insights relevant to human health^14^. Early canine microbiome studies, predominantly using short-read sequencing, revealed how diet, host genetics, and environment influence gut communities^15^. More recently, long-read platforms have enabled near-complete bacterial genome assemblies from canine feces, providing deeper functional and taxonomic resolution^16^. Despite this relevance, research on the canine gut microbiome remains hampered by methodological inconsistencies in DNA isolation, library preparation, and sequencing workflows, which limit reproducibility and cross-study comparability. Lifelong monotonous diets make nutrient absorption, diet-linked phenotypes (e.g., obesity), and gastrointestinal disorders particularly significant in dogs^13,17^. Moreover, a significant proportion of the microbial taxa in the dog gut remains uncharacterized^13^, making it more challenging to establish targeted microbial biomarkers or therapeutic strategies for canine health. Additionally, understanding how the canine microbiome relates to that of pet owners could provide valuable insights into shared microbial influences and potential cross-species microbial exchange. Nevertheless, key questions remain about how best to integrate extraction kits, primer designs, and sequencing technologies to comprehensively characterize the canine gut microbiome.

The dataset includes three components reflecting commonly used methodological dimensions in microbiome studies: (i) comparison of DNA extraction approaches, (ii) evaluation of sequencing strategies, and (iii) assessment of primer-associated variability.

- Multi-Platform, Single-Canine Fecal Evaluation: A single dog’s fecal sample was analyzed using both short-read (Illumina NovaSeq) and long-read (Oxford Nanopore MinION) sequencing platforms.
- Comparison of Two DNA Extraction Methods in Forty Canine Fecal Samples: Two DNA extraction kits were compared — Zymo High-Molecular-Weight (ZHMW; optimized for long-read sequencing) and Zymo MagBead (ZMB; routine magnetic bead workflow) — across 40 samples from a longitudinal familial cohort.
- Primer Comparison in Synthetic and Biological Samples: Three 16S primer configurations were evaluated: (A) standard ONT (V1–V9), (B) a PacBio-derived full-length 16S primer set, and (C) modified ONT (MONT) primers containing degenerate bases. These were applied to mock communities (Zymo D6300, D6331, D6323) and fecal samples from human and canine donors, all sequenced on the Oxford Nanopore MinION platform.

## Methods

**Fig. 1:**
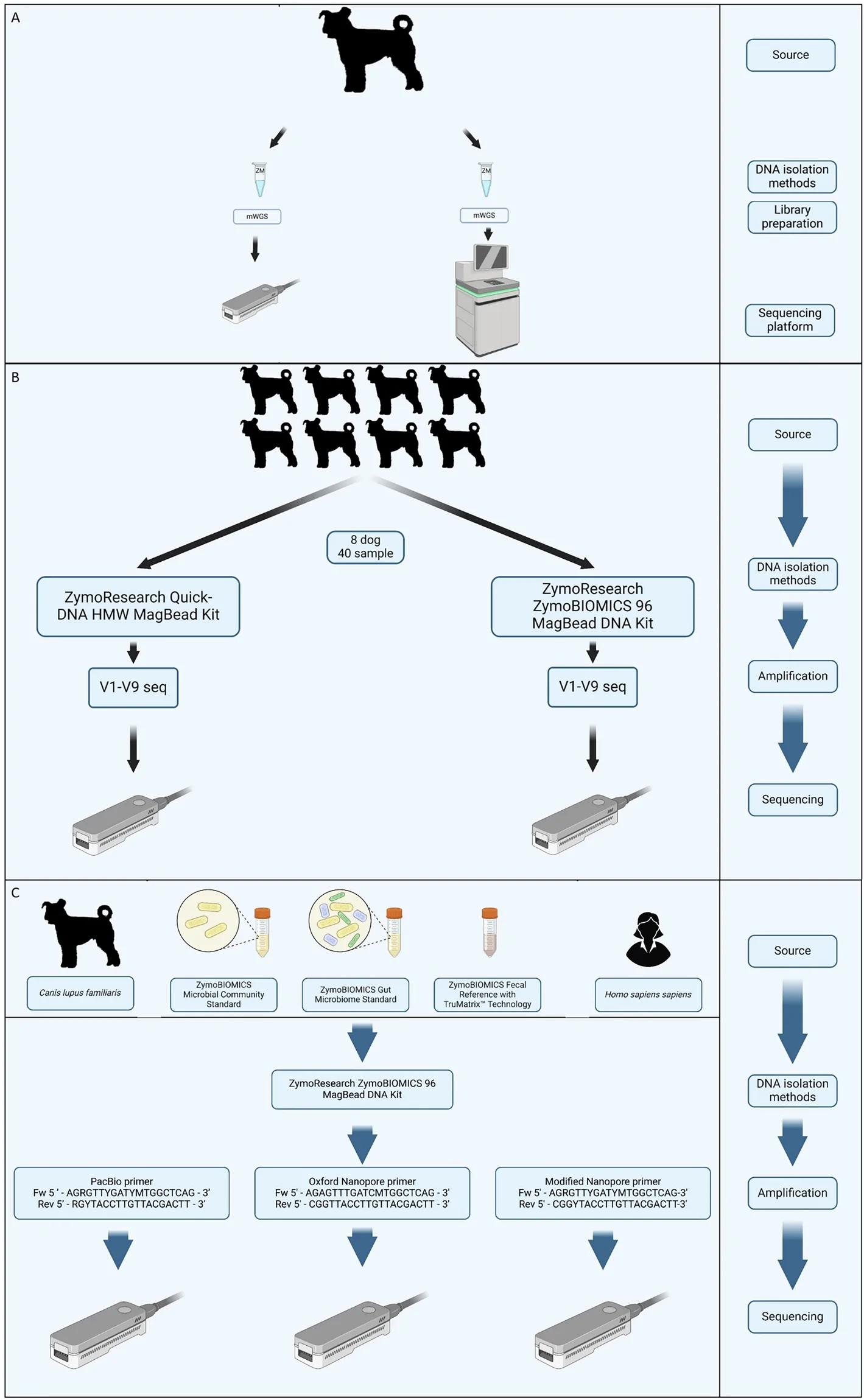
Overview of study design and experimental workflows. (**A**) Schematic representation of the single-sample multi-platform dataset. A fecal sample from a single dog (Canis lupus familiaris) was processed for metagenomic whole-genome sequencing (mWGS) using both Oxford Nanopore Technologies (ONT) MinION (long-read sequencing) and Illumina NovaSeq (short-read sequencing) platforms. (**B**) Design of the DNA extraction comparison dataset. Fecal samples collected from eight dogs (40 samples in total) were split prior to DNA isolation and processed in parallel using two extraction methods: the ZymoBIOMICS™ 96 MagBead DNA Kit (ZMB) and the Quick-DNA™ HMW MagBead Kit (ZHMW). Following extraction, full-length 16S rRNA gene (V1–V9 region) libraries were prepared and sequenced using the ONT MinION platform. (**C**) Overview of the primer comparison dataset. Three types of input material were used: canine fecal samples, synthetic microbial community standards, and a human fecal sample (Homo sapiens). DNA was isolated using the ZymoBIOMICS™ 96 MagBead DNA Kit, followed by amplification of the full-length 16S rRNA gene (V1–V9) using three primer configurations: standard ONT primers, modified ONT (MONT) primers, and a PacBio-derived (PB) primer set. Libraries were sequenced on the ONT MinION platform.

The study workflow is detailed in Fig. 1. DNA isolation and library preparation followed the manufacturers’ protocols strictly. Full methodological details are provided below.

### Sample sources

The sequencing yields and basic metadata for the three datasets are summarized in Table 1. The details about the dogs used in the study can be found in Supplementary Table S1.

**Table 1:** Basic information about the datasets.

Study phase	Source	Sample type	Dog No.	# of samples	# of replicates	DNA isolation method	Type of library	Platform	Gigabases (raw)	ENA Project
1. Single-Canine Multi-Platform Dataset	This study	Single dog, 1 sample	M0	2	1	ZMB	WGS	NovaSeq	5.41	PRJEB75753
	This study		M0	2	1	ZMB	WGS	MinION	3.78	PRJEB75753
2. Zymo MagBead and HMW Kit Comparison in Canine Fecal Metagenomics	This study	Eight dogs	F1-F4 & M1-M4	total of 40	3 × 3	ZHMW & ZMB	V1-V9	MinION	43.45	PRJEB82097
3. V1–V9 Primer Comparison Dataset on Synthetic and Biological Samples	This study	Single dog, 1 sample	F5	1	3 × 3	ZMB	V1-V9	MinION	22.61	PRJEB85420
	This study	Single human sample	NA	1	3 × 3	ZMB	V1-V9	MinION		PRJEB85714
	This study	MCS	NA	1	3 × 3	ZMB	V1-V9	MinION		PRJEB85420
	This study	GMS	NA	1	3 × 3	ZMB	V1-V9	MinION		PRJEB85420
	This study	FR	NA	1	3 × 3	ZMB	V1-V9	MinION		PRJEB85420

#### Single-canine multi-platform dataset

A single fecal sample was collected from an adult *Canis lupus familiaris* (Dog_M0). The dog was privately owned, and the feces were obtained shortly after defecation to minimize environmental contamination. The collected material was immediately stored at −20 °C, then transferred to −80 °C within 24 h. The sample (1.0 g) was subdivided for metagenomic whole-genome sequencing on both ONT MinION and Illumina NovaSeq platforms. Due to the limited sample size, homogenization was not performed. This approach aligns with the findings of Liang *et al*.^17^, who demonstrated that while homogenization is essential for metabolomic analyses, it is not necessary for microbiome studies. Supplementary Table S2 contains information about the sequenced datasets from this sample.

#### DNA Extraction comparison on forty dog fecal samples dataset

Fecal samples were collected from eight co-housed adult dogs from the Serteperti Pumi Kennel (Dány, Hungary) over a one-year period (the Serteperti cohort). Each dog produced multiple time-point samples, and 40 of these were selected for a direct comparison of two DNA isolation methods (Zymo High-Molecular-Weight vs. MagBead). Immediately after natural defecation, ~1–3 g of feces was scooped into sterile containers, frozen promptly (−20 °C), and transferred to the laboratory for further storage (−80 °C). All dogs were housed under consistent husbandry conditions. Female dogs were kept both indoors and outdoors, whereas male dogs were housed exclusively outdoors. All dogs were fed a commercial dry food formulated for adult dogs (Gemon Lamb & Rice Adult, Monge, Italy). Supplementary Table S3 contains the read counts and barcodes for the sequencing runs. Supplementary Fig. S1 shows the familial relationships among the dogs from the Serteperti cohort.

#### V1–V9 Primer comparison dataset on synthetic and biological samples

To evaluate three distinct 16S rRNA primer sets (ONT, MONT, and PB), we utilized:

1. *Three Synthetic Mixtures*:
  ZymoBIOMICS® Microbial Community Standard (Cat. #: D6300): This mock community comprises eight bacterial and two fungal strains, including both easy-to-lyse Gram-negative bacteria and tough-to-lyse Gram-positive bacteria and yeasts.
  ZymoBIOMICS® Gut Microbiome Standard (Cat. #: D6331): This standard featured a more complex mock community comprised of 21 different strains, designed to mimic the human gut microbiome.
  ZymoBIOMICS® Fecal Reference with TruMatrix™ Technology (Cat. #: D6323): This reference material contains standardized human stool components, providing a complex microbial community representative of the human gut microbiome.
  The reference composition used in our analyses was deposited in our GitHub repository https://github.com/Balays/CaniMeta/tree/main/standards.
2. *Two Biological Samples:*
  One fecal sample from a female volunteer (noninvasive, anonymous donation) from Hungary (age between 50–55 years).
  One canine fecal sample (F5) collected under the same conditions as the others from Ajka-Padragkút, Hungary.

All mock standards were obtained from Zymo Research (Irvine, CA, USA). The biological fecal samples were placed on ice immediately after collection, then frozen at −20 °C and later shipped to the lab for long-term storage (−80 °C) and processing.

Each sample (synthetic or biological) was subjected to the three primer sets targeting the full 16S V1–V9 region, followed Oxford Nanopore (MinION). By sequencing on the Oxford Nanopore MinION platform.

### Ethics

This study was conducted in accordance with the principles of the Declaration of Helsinki. Ethical approval was obtained from the Medical Research Council, Budapest, Hungary, under the accession number BMEÜ/725-1 /2022/EKU. Written informed consent was obtained from the volunteer participant and the dog owners for sample collection and data publication.

### DNA purification

DNA extraction protocols were applied according to the specific study component as follows:

1. Single-Canine Fecal Sample (Part 1):

- Zymo MagBead DNA isolation kit was used following the manufacturer’s recommended input of 200 mg feces.

2) Forty Dog Fecal Samples (Zymo HMW vs. MagBead, Part 2):

- Each of the 40 targeted fecal samples was split into two aliquots. One aliquot underwent ZHMW extraction (using 100 mg of canine feces), while the other used the MagBead protocol at 200 mg, following the standard manufacturer’s recommendations.

3) Mock Communities (MCS) and Additional Samples (Part 3):

- In the evaluation of different 16S primer sets, 75 μL of the Zymo Microbial Community Standard (MCS) was used as input for the Zymo MagBead DNA isolation kit, adhering to the manufacturer’s protocol.

All extractions were performed strictly according to each kit’s instructions, with the noted exceptions (100 mg vs. 200 mg feces for Zymo kits).

#### Zymo Research Quick-DNA™ HMW MagBead Kit

The fecal sample was resuspended in 200 µl of DNA/RNA Shield™, followed by a 5-minute incubation at room temperature (20–30 °C) on a tube rotator. Then, 33 µl of MagBinding Beads were added to each sample, mixed thoroughly, and shaken for 10 minutes. The sample was placed on a magnetic stand to allow the beads to separate clearly from the solution, and the supernatant was then removed. For the washing process, 500 µl of Quick-DNA™ MagBinding Buffer was added, the beads were resuspended, and the mixture was shaken for 5 minutes. The sample was placed back on the magnetic stand to discard the supernatant. Subsequently, 500 µl of DNA Pre-Wash Buffer was added and the beads were again resuspended. After returning the sample to the magnetic stand and discarding the supernatant, 900 µl of g-DNA Wash Buffer was added, mixed, and transferred to a new tube. The magnetic stand facilitated the separation of the beads from the solution, and the supernatant was discarded. This washing step was repeated once more. The beads were allowed to air dry for 20 minutes. In the elution phase, 50 µl of DNA Elution Buffer was added to the beads. Following mixing, the solution was left to incubate at room temperature for 5 minutes. The sample was then placed on the magnetic stand once more to separate the beads from the solution. The eluted DNA was carefully transferred to a new tube and stored at −20 °C for later analysis.

#### Zymo Research ZymoBIOMICS™ 96 MagBead DNA Kit - Dx

Samples were added to the Zymo Research (ZR) BashingBead™ Lysis Tubes and 750 µl of ZymoBIOMICS™ Lysis Solution was also added. The mixture was then centrifuged at ≥10,000 × g for 1 minute. Two hundred µl of the supernatant were transferred to new tubes, and then 600 µl of ZymoBIOMICS™ MagBinding Buffer was added. Twenty-five µl of ZymoBIOMICS™ MagBinding Beads were dispensed into each tube, and the contents were mixed on a shaker plate for 10 minutes. The tubes were then placed on a magnetic stand until the beads settled, after which the supernatant was aspirated and discarded. Tubes were removed from the magnet, and 500 µl of ZymoBIOMICS™ MagBinding Buffer was added and mixed well on a shaker plate for 1 minute. The process of placing the tubes on the magnetic stand to settle the beads and discarding the supernatant was repeated. Subsequently, 500 µl of ZymoBIOMICS™ MagWash 1 was added to each tube and shaken for 1 minute. After placing the tubes on the magnet again and discarding the supernatant, 900 µl of ZymoBIOMICS™ MagWash 2 was added and mixed thoroughly for 1 minute. The tubes were placed on the magnet until the beads settled and the supernatant was again discarded. The final washing step was repeated. The samples were heated on a thermal block at 55 °C until the beads dried, which took approximately 10 minutes. Then, 50 µl of ZymoBIOMICS™ DNase/RNase-Free Water was added to each tube and the beads were resuspended. The mixture was shaken for 10 minutes, then transferred to a magnetic stand for 2-3 minutes until the beads settled. The supernatant containing the eluted DNA was transferred to a clean elution tube. The eluted DNA samples were stored at −20 °C for future use.

### Library preparation

Depending on the sequencing strategy – metagenomic whole-genome sequencing (mWGS) or full V1–V9 16S rRNA amplicon sequencing – different library preparation kits and sequencing platforms were employed:

1. Metagenomic Whole-Genome Sequencing (mWGS)

- Illumina Platforms (NovaSeq): Libraries were constructed using the xGen™ DNA Library Prep Kit (IDT). These short-read libraries targeted ~300–500 bp inserts and underwent paired-end sequencing.
- Oxford Nanopore Technologies (ONT, MinION): For long-read mWGS, we used the ONT Native Barcoding Kit 24 V14, enabling multiplexed sequencing of canine fecal DNA on the MinION device.

2) 16S rRNA Gene Amplicon Sequencing (Full V1–V9 Region)

- Library preparation was performed according to manufacturer protocols with modifications as described below. Routine quality control checks (Qubit quantification and TapeStation or Bioanalyzer analysis) were used to confirm DNA concentration and fragment size distributions.

#### IDT xGen^TM^ DNA Lib Prep Kit

DNA samples (100 ng) were adjusted to a final volume of 19.5 µL using Low EDTA TE buffer. Components including 3 µL of Buffer K1, 1.5 µL of Reagent K2, and 6 µL of Enzyme K3 were added to each sample. The mixture was processed in a thermal cycler following the manufacturer’s Enzymatic Prep protocol, with inactivation at 65 °C for 30 minutes and the thermal cycler lid set to 70 °C. Post-reaction, samples were stored at 4 °C. The fragmented DNA underwent ligation preparation by adding 12 µL of Buffer W1, 4 µL of Enzyme W3, 5 µL of stubby adapter Reagent W5, and 9 µL of Low EDTA TE to each sample, making up a 30 µL Ligation Master Mix. Ligation was conducted at 20 °C for 20 minutes. Post-ligation, the samples were cleaned up using SPRIselect beads. Initially, 48 µL of beads were added to each sample at room temperature, mixed by vortexing for 5 seconds, and briefly centrifuged. The samples were incubated for 5 minutes at room temperature and then placed on a magnetic rack until the supernatant cleared. The supernatant was discarded, and the beads were washed twice with 180 µL of freshly prepared 80% ethanol, each wash followed by a brief incubation and removal of ethanol. After drying, 20 µL of Low EDTA TE was added, and the beads were resuspended. DNA was eluted into a new tube after a 2-minute incubation at room temperature. PCR amplification was then performed on the eluted DNA. The PCR setup involved an initial denaturation at 98 °C for 45 seconds, followed by cycles of denaturation at 98 °C for 15 seconds, annealing at 60 °C for 30 seconds, and extension at 72 °C for 30 seconds, concluding with a final extension at 72 °C for 1 minute. A final post-PCR cleanup was conducted similar to the initial cleanup steps, using 48 µL of beads per sample and repeating the washing steps with 80% ethanol. The final DNA was resuspended in 21 µL of Low EDTA TE and transferred to a clean tube for storage or further analysis.

#### Oxford Nanopore Technologies Ligation Sequencing DNA V14 Kit

One μg of genomic DNA was adjusted to a final volume of 48 μl with nuclease-free water. The following reagents were then added: 3.5 μl NEBNext FFPE DNA Repair Buffer, 3.5 μl Ultra II End-prep Reaction Buffer, 3 μl Ultra II End-prep Enzyme Mix, and 2 μl NEBNext FFPE DNA Repair Mix (all from New England Biolabs, Ipswich, MA, USA). The mixture was incubated in a thermal cycler at 20 °C for 5 minutes then 65 °C for 5 minutes. After the incubation, AMPure XP beads were resuspended and 60 μl of the bead suspension was added to the end-prep reaction. The sample was incubated for 5 minutes on a Hula mixer at room temperature then washed with 80% ethanol. After the end-repair, the DNA was eluted in 61 μl of nuclease-free water. For adapter ligation, 5 μl Ligation Adapter, 10 μl NEBNext Quick T4 DNA Ligase, and 25 μl Ligation Buffer was added to the sample. After 10 minutes of incubation at RT, 40 μl of resuspended AMPure XP Beads were added to the reaction. The mixture was incubated on a Hula mixer for 10 minutes, then washed with Short Fragment Buffer (SFB), and the DNA was eluted in 13 μl of Elution Buffer.

#### Combining the ONT Rapid 16S Barcoding Kit and Native Barcoding Kit for Custom Primer Sequences

Because the Oxford Nanopore Technologies Rapid Sequencing 16S Barcoding Kit incorporates barcoded primers, a combined protocol was employed to enable the attachment of sequencing barcodes and adapters to the custom or modified primer sequences.

Reactions were prepared in 0.2 mL thin-walled PCR tubes by combining 5 µL nuclease-free water, 10 µL input DNA (10 ng), and 25 µL LongAmp Hot Start Taq 2 × Master Mix (New England Biolabs, USA), following the Rapid Sequencing Amplicons protocol (SQK-16S024). Three primer sets were used: the standard ONT primer (forward: 5′-AGAGTTTGATCMTGGCTCAG-3′, reverse: 5′-CGGTTACCTTGTTACGACTT-3′), the modified ONT (MONT) primer (forward: 5′-AGRGTTYGATYMTGGCTCAG-3′, reverse: 5′-CGGYTACCTTGTTACGACTT-3′), and a PacBio-derived full-length 16S degenerate primer set (forward: 5′-AGRGTTYGATYMTGGCTCAG-3′, reverse: 5′-RGYTACCTTGTTACGACTT-3′). Note that the MONT and PacBio-derived primer sets share an identical forward sequence and differ only in the reverse primer. Ten µL of each primer pair was added to each reaction and mixed thoroughly. In a thermal cycler, the reaction was amplified using the following cycling conditions: initial denaturation 1 min (95 °C, 1 cycle), denaturation 20 secs (95 °C, 25 cycles), annealing 30 secs (55 °C, 25 cycles), extension 2 mins (65 °C, 25 cycles), final extension 5 mins (65 °C, 1 cycle), and hold on 4 °C. On a magnetic rack, the PCR products were cleaned using AMPure XP beads, washed with 70% ethanol, and eluted in 10 µL nuclease-free water. From the following step, we used the Native Barcoding Kit.

We used 200 fmol amplicon DNA and made up each sample to 12.5 μl with nuclease-free water, then added Ultra II End-prep Reaction Buffer and Ultra II End-prep Enzyme Mix from New England Biolabs (NEB). The mixture was well mixed, briefly centrifuged, and then incubated in a thermal cycler at 20 °C for 5 minutes followed by 65 °C for 5 minutes. Then, 0.75 μl of sample was transferred to a new strip. The NEB Blunt/TA Ligase Master Mix was prepared as per the manufacturer’s instructions and kept on ice. After thawing and mixing, the reagents were briefly centrifuged. Native Barcodes (NB01-45) were prepared and kept on ice.

To each sample, a selected Native Barcode, Blunt/TA Ligase Master Mix and Nuclease-free water were added. The mixture was then incubated for 20 minutes at room temperature. After that EDTA was added to stop the reaction and the barcoded samples were pooled.

The pooled sample was mixed with 0.4X volume of AMPure XP Beads and incubated on a Hula mixer for 10 minutes. After washing with 80% ethanol, DNA was eluted in 31 μl of nuclease-free water, with intermittent flicking at 37 °C to facilitate elution.

For adapter ligation, the NEBNext Quick Ligation Reaction Module was prepared according to the manufacturer’s instructions. The pooled barcoded sample was combined with Native Adapter, Quick T4 DNA Ligase, and NEBNext Quick Ligation Reaction Buffer (5X). Following ligation, 20 μl of AMPure XP Beads were added to the reaction. The mixture was incubated on a Hula mixer for 10 minutes, washed with Short Fragment Buffer (SFB), and the DNA was eluted in 15 μl of Elution Buffer after a 10-minute incubation at 37 °C. The final DNA library was prepared by adjusting the concentration to the desired level with Elution Buffer for sequencing.

#### Oxford Nanopore Technologies Rapid Sequencing 16S Barcoding Kit

Ten ng of high molecular weight genomic DNA, in a 10 µl volume, was utilized for library preparation from the canine samples. The input DNA was combined with 14 µl of nuclease-free water (Invitrogen), 1 µl of 16S Barcode (1 µM; Supplementary Tables S3, S4), and 25 µl of LongAmp Taq 2X master mix (New England Biolabs). The PCR amplification of the samples was performed starting with an initial denaturation at 95 °C for 1 minute. This was followed by 25 cycles of denaturation at 95 °C for 20 seconds, annealing at 55 °C for 30 seconds, and extension at 65 °C for 2 minutes. A final extension was carried out at 65 °C for 5 minutes to complete the reaction. Post-amplification, the DNA samples were transferred to clean 1.5 ml Eppendorf DNA LoBind tubes and mixed with 30 µl of resuspended AMPure XP beads. The samples were then agitated on a Hula mixer for 5 minutes at room temperature. Afterward, the tubes were placed on a magnetic rack, and the supernatant was discarded. The beads were washed with 200 µl of freshly prepared 70% ethanol, the ethanol was removed, and the washing step was repeated. Following air drying of the beads, the tubes were removed from the magnet, and the beads were resuspended in 10 µl of 10 mM Tris-HCl pH 8.0 with 50 mM NaCl. After a 2-minute incubation at room temperature, the tubes were placed back on the magnet. Ten µl of the clean supernatant, containing the ONT libraries, was transferred to a new Eppendorf DNA LoBind tube. The barcoded libraries were then pooled in an equal molar ratio, and 1 µl of RAP was added. The mixture was incubated for 5 minutes at room temperature. Subsequently, one hundred femtomoles of the DNA libraries were loaded onto a MinION flow cell for sequencing.

#### Quality and Quantity Check

After the DNA isolation and library preparation methods (Oxford Nanopore, Illumina NovaSeq), the genomic DNA samples were quantified and assessed for quality. DNA concentrations were measured using the Invitrogen™ Qubit™ 4 Fluorometer with the Qubit™ 1X dsDNA High Sensitivity (HS) kit. For quality assessment, the Agilent Technologies 4150 TapeStation System was used. The Genomic DNA ScreenTape was applied for genomic DNA and Oxford Nanopore WGS evaluation, the D5000 ScreenTape for ONT V1-V9, and the D1000 ScreenTape for V region amplicon libraries.

### Sequencing

Sequencing was performed using Illumina NovaSeq and Oxford Nanopore Technologies MinION platforms, depending on the dataset. The single-sample multi-platform dataset (Part 1) included both Illumina NovaSeq and Oxford Nanopore sequencing, whereas the DNA extraction comparison dataset (Part 2) and the V1–V9 primer comparison dataset (Part 3) were sequenced using Oxford Nanopore MinION. Nanopore sequencing was carried out using different flow cell chemistries: R10 flow cells were used for Parts 1 and 3, and R9 flow cells for Part 2. Libraries were loaded and sequenced according to the manufacturers’ recommendations under standard MinION operating conditions^18,19^.

### Bioinformatics

#### Single-canine multi-platform dataset

##### Illumina NovaSeq whole-genome shotgun data

Paired-end Illumina NovaSeq reads were processed using fastp (v0.24.0) for adapter trimming, quality filtering, and read correction. Processing included removal of low-quality bases (Phred score <25), polyG trimming, read deduplication, and merging of overlapping read pairs. Base trimming from read ends was also applied (12 bases from the 5′ end and 3 bases from the 3′ end of both reads). Additional filtering steps included error correction and removal of low-quality or failed reads. The following exact parameters were used:–trim_front1 12–trim_front2 12–trim_tail1 3–trim_tail2 3 -q 25–dedup–merge–correction–trim_poly_g.

##### Oxford Nanopore MinION data

Nanopore sequencing data were basecalled using Dorado (Oxford Nanopore Technologies, v0.8.3)^19^ with super-accurate models appropriate for the respective flow cell chemistries (R9 for Part 2 and R10 for Parts 1 and 3). Reads were demultiplexed based on barcode sequences using ONT software tools, and resulting FASTQ files were used for downstream processing and were deposited under the appropiate project accession.

##### Taxonomic classification

Taxonomic classification of both Illumina and nanopore datasets was performed using sourmash (v4.8.14)^20^ with a comprehensive reference database including bacterial, archaeal, and eukaryotic genomes. Presence–absence information derived from sourmash classifications was used to assess overlap of detected taxa between sequencing platforms at the phylum level.

#### DNA Extraction comparison on forty dog fecal samples dataset

Nanopore sequencing data generated from paired DNA extractions (ZHMW and ZMB kits) were basecalled using Dorado with the super-accurate model and demultiplexed by barcode. Sequencing performance metrics, including read counts, read lengths, and per-read quality scores, were evaluated to assess reproducibility between extraction methods. No downstream taxonomic analyses from this dataset are included in the present study.

#### V1–V9 Primer comparison dataset on synthetic and biological samples

Sequencing data generated using alternative primer configurations were basecalled using Dorado with models appropriate for R10 flow cells and demultiplexed by barcode.

Oxford Nanopore reads were basecalled and demultiplexed prior to downstream analysis. Full-length 16S rRNA gene reads were length-filtered to retain near full-length amplicons (1500–1800 bp).

Taxonomic classification was performed using Emu (v3.5.0)^21^, which applies an expectation–maximization framework for species-level profiling of long-read 16S data. The emu abundance command was used with parameters specifying ONT sequencing data (–type map-ont), a minimum abundance threshold (–min-abundance), and multithreading. Output files included abundance tables, read assignments, and unclassified reads.

Species-level presence–absence information was used to evaluate detection of expected taxa in the mock communities and fecal reference at the species and genus levels.

Presence–absence information derived from Emu classifications was also used to summarize overlap of detected taxa between the fecal reference and the biological samples at the species level.

##### Downstream Data Processing and Visualization

Sequencing statistics and taxonomic classification outputs were processed in the R statistical environment (R v4.4) using custom scripts. Data handling relied primarily on the data.table package^22^ for efficient manipulation of large datasets.

Sequencing quality summaries included distributions of read length and per-read quality scores, as well as total read counts per sample. Data visualization was performed using ggplot2^23^ and ggpubr^24^. For large datasets, rasterized point layers were used to improve rendering efficiency while preserving data density.

Overlap analyses were based on presence–absence matrices derived from taxonomic classifications. Euler diagrams were generated using eulerr to illustrate shared and dataset-specific taxa^25,26^. Taxonomic data were handled mainly using the phyloseq R package^27^.

## Data Records

The dataset is available at https://github.com/Balays/CaniMeta. The repository is organized into three study-specific directories, 1_Dog.M0_WGS, 2_40dogs_ZHMW_vs_ZMB, and 3_Primer_comparison, together with shared reference and workflow directories, including ENA, databases, standards, and scripts. The main repository-level notebook, CaniMeta.Rmd, serves as the central workflow for sequence download from ENA, formatting of processed QC tables, and generation of the QC report across the study datasets.

The shared ENA directory contains files associated with sequence retrieval and run-level records from the European Nucleotide Archive. The databases directory contains the reference taxonomy resources used for taxonomic assignment, including the EMU and sourmash reference databases. The standards directory contains the reference composition files and associated documentation for the Zymo mock communities used as controls. The scripts directory contains the helper shell and R scripts used for sequence download, read-statistic generation, compression, and QC reporting.

Within 1_Dog.M0_WGS, files relate to the Dog.M0 whole-genome sequencing comparison and include processed QC tables, taxonomic-analysis outputs, and figure files. Within 2_40dogs_ZHMW_vs_ZMB, files relate to the 40-dog comparison of the two DNA isolation methods and include metadata tables, processed read-level and summary tables, EMU-based taxonomic outputs, and manuscript figures. Within 3_Primer_comparison, files relate to the primer-comparison analyses and include processed QC tables, metadata, taxonomic comparison outputs for the different mock and biological sample sets, and the corresponding analysis notebooks and figures.

The sequencing yields and basic metadata for these datasets are summarized in Table 1. All datasets are provided in standard formats and can be accessed directly via the repository interfaces.

## Technical Validation

### Single-canine multi-platform dataset

To evaluate technical reliability, sequencing performance metrics were assessed for the Dog_M0 sample generated using Illumina NovaSeq and Oxford Nanopore MinION platforms.

#### Sequencing performance

Basic sequencing statistics for each dataset are provided in Supplementary Table S2. Illumina NovaSeq sequencing produced approximately 17.9 million paired-end reads with mean per-read quality scores around Q36, whereas Oxford Nanopore MinION sequencing generated 2,956,194reads with mean per-read quality scores around Q30 and a mean length of 1,280 bp. These values are consistent with the expected performance characteristics of the respective sequencing platforms.

**Fig. 2:**
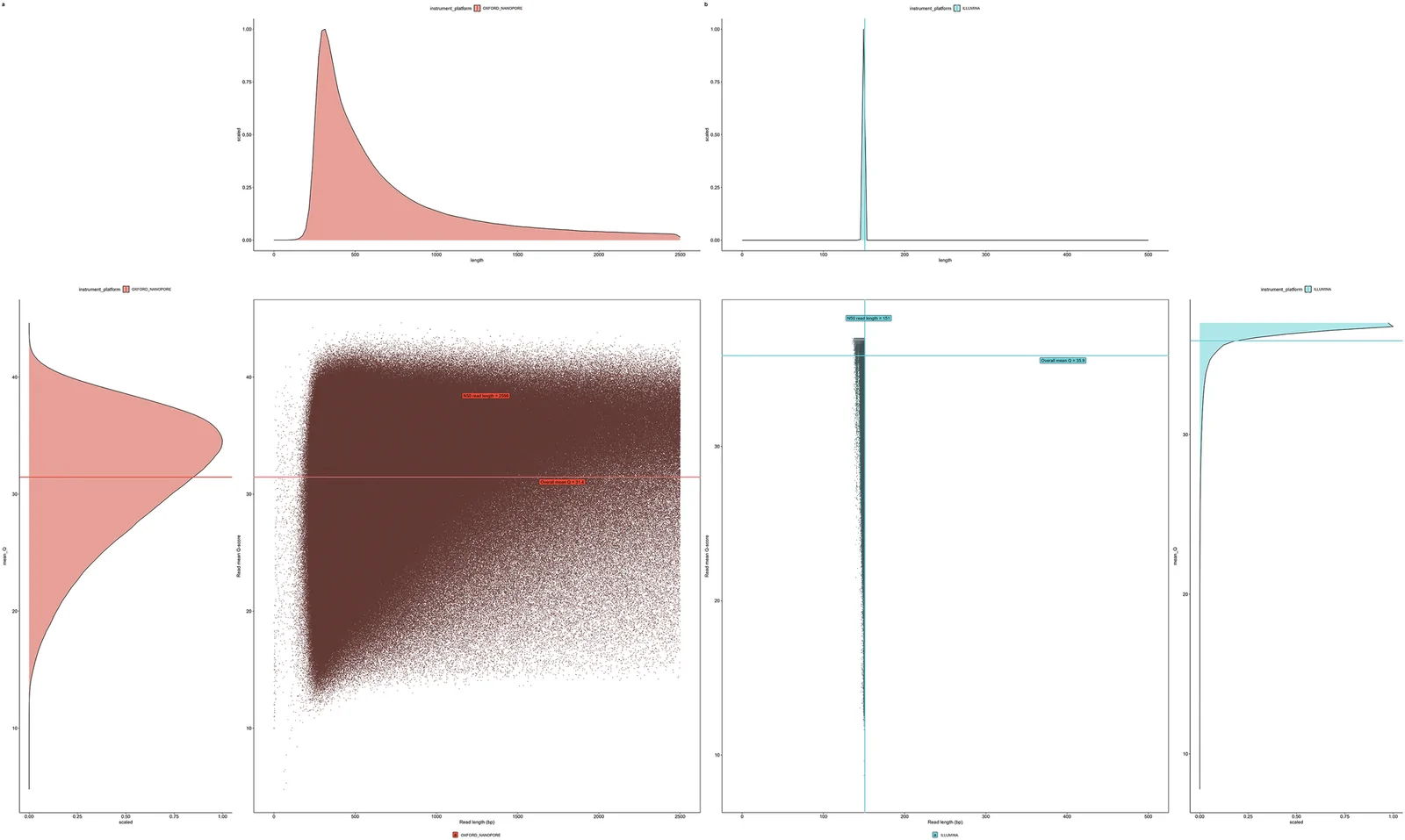
Sequencing characteristics of the Dog_M0 sample. (**a**) Read-level quality control for Oxford Nanopore MinION sequencing. The central scatter plot shows read length versus mean per-read quality score, with marginal density plots summarizing the distributions of read length and quality. The vertical line indicates the N50 read length, and the horizontal line indicates the overall mean read quality score. Nanopore reads span a broad length range extending to approximately 2.5 kb and exhibit the expected distribution of per-read quality scores. (**b**) Read-level quality control for Illumina NovaSeq sequencing. The central scatter plot shows read length versus mean per-read quality score, with marginal density plots summarizing the distributions of read length and quality. The vertical line indicates the N50 read length, and the horizontal line indicates the overall mean read quality score. Illumina reads are tightly distributed around ~150 bp and show a narrow quality-score distribution consistent with short-read sequencing.

Read-level quality control metrics are shown separately for the two platforms in Fig. 2. For each platform, scatter plots of read length versus mean per-read quality score are accompanied by marginal density plots summarizing the distributions of read length and quality. Vertical lines indicate the N50 read length, and horizontal lines indicate the overall mean read quality score. Illumina reads are tightly distributed around ~150 bp (Fig. 2b), whereas nanopore reads span a broader range and extend to approximately 2.5 kb (Fig. 2a). Illumina data show a narrow quality-score distribution, while nanopore reads display a broader distribution centered around the expected range for MinION sequencing.

Because quality scores are derived from platform-specific basecalling and error models, Q-score distributions should be interpreted as within-platform quality summaries rather than as directly comparable measures across technologies.

#### Overlap of detected taxa

Overlap analysis of detected taxa between the two sequencing datasets is shown in Supplementary Fig. S2. At the phylum level, 73 taxa were detected in both datasets, while 15 were detected only in the nanopore dataset and 7 only in the NovaSeq dataset.

### DNA Extraction comparison on forty dog fecal samples dataset

To assess reproducibility of DNA extraction workflows, fecal samples from individual dogs were processed in parallel using two protocols: the Zymo MagBead kit (ZMB) and the Zymo High Molecular Weight kit (ZHMW). Each sample was split prior to extraction, enabling paired comparisons of sequencing output.

#### Sequencing yield and read quality

**Fig. 3:**
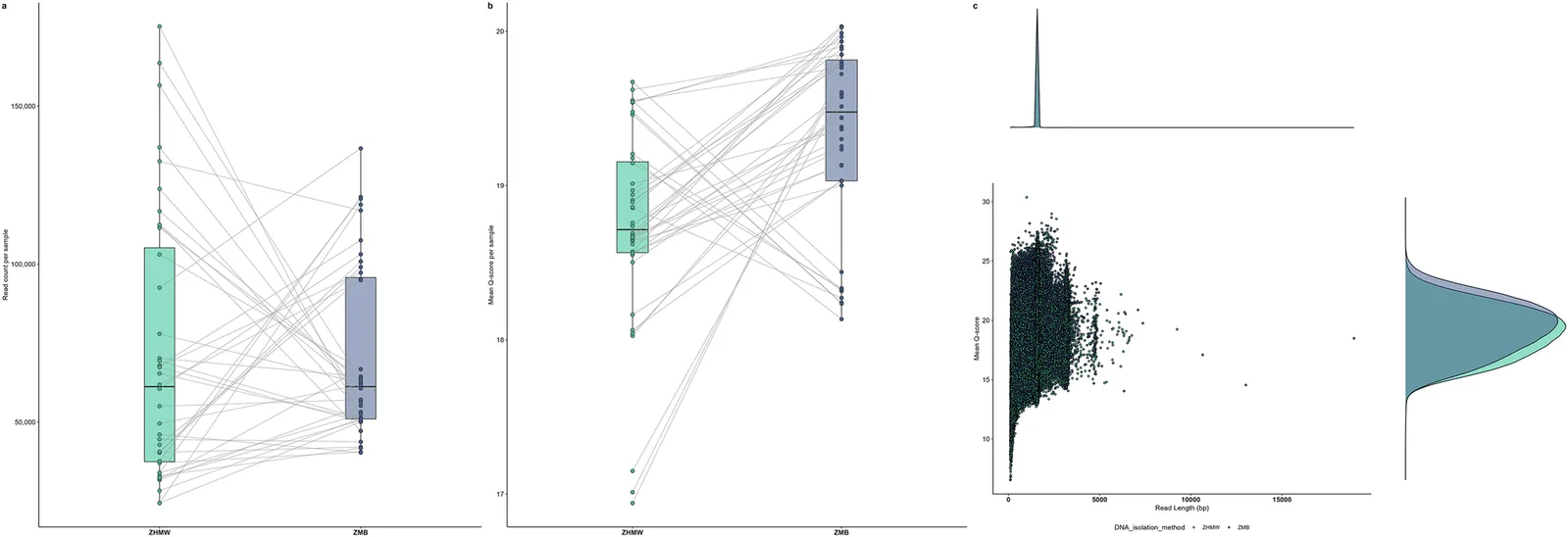
Paired DNA extraction reproducibility. (**a**) Total number of nanopore reads generated per sample following DNA extraction using ZHMW and ZMB protocols. Points represent individual samples; grey lines connect paired extractions from the same biological sample. (**b**) Mean per-read Phred quality scores for nanopore sequencing reads obtained from each extraction method. (**c**) Scatter plot of read length versus mean per-read quality score for nanopore sequencing reads obtained from the ZHMW and ZMB extraction methods. Marginal density plots depict the distributions of read length and quality. The dominant peak near ~1.5–1.6 kb corresponds to full-length V1–V9 16S rRNA gene amplicons.

Nanopore sequencing generated comparable numbers of reads across extraction methods (Fig. 3a). Both protocols produced on the order of tens of thousands of reads per sample, with variability observed among individual samples but no systematic failures associated with either workflow. Mean per-read Phred quality scores were also similar between protocols (Fig. 3b). Quality values for both methods fell within the expected range for nanopore amplicon sequencing. Figure 3c shows the read mean quality versus length distribution for the two methods across the whole dataset, while Supplementary Fig. S3 shows these distributions for each sample separately. These observations indicate that both extraction procedures produced DNA suitable for MinION sequencing and generated datasets of comparable technical quality.

### V1–V9 Primer comparison dataset on synthetic and biological samples

#### Taxonomic detection metrics

**Fig. 4:**
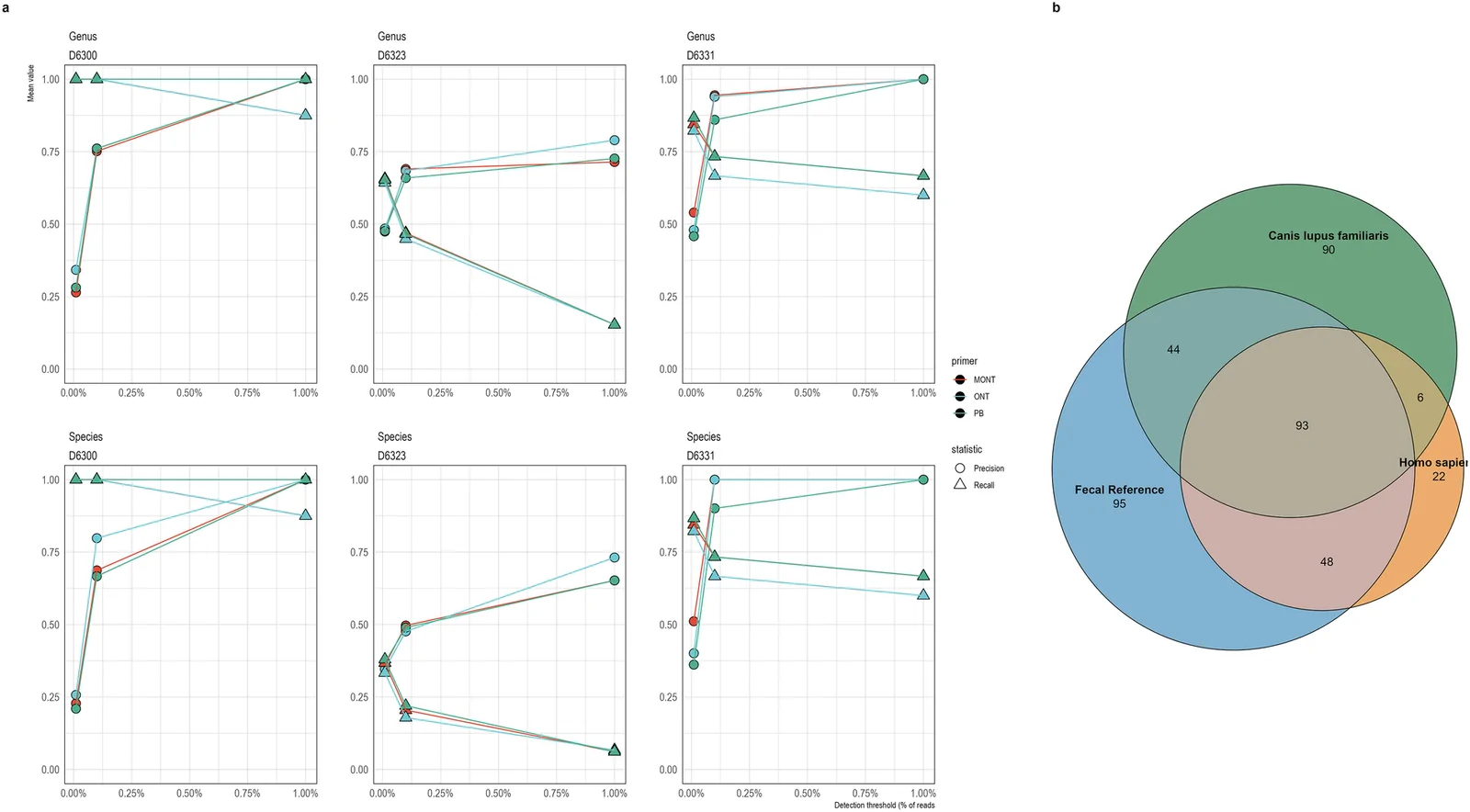
Reference material metrics and cross-dataset overlap. (**a**) Precision and recall values calculated for two synthetic microbial community standards (D6300, D6331) and one fecal reference community (D6323) at genus and species levels across three detection thresholds (0.01%, 0.1%, and 1% of reads) and primer configurations. (**b**) Euler diagram showing species-level overlap among the fecal reference community (D6323), a human fecal sample, and a canine fecal sample. Values indicate the number of species detected exclusively in each dataset and those shared between datasets or across all three.

Taxonomic detection metrics were calculated using two synthetic microbial community standards (D6300 and D6331) and one fecal reference community derived from a human sample (D6323). Precision and recall were computed at genus and species levels across three detection thresholds (0.01%, 0.1%, and 1% of reads) for libraries generated using ONT, MONT, and PB primer configurations (Fig. 4a).

#### Species overlap across datasets

Species-level overlap among the fecal reference community (D6323), a human fecal sample, and a canine fecal sample was evaluated using presence–absence information (Fig. 4b). Shared and dataset-specific detections were observed across all three sample types, and the detailed overlap structure is shown in the Euler diagram.

#### Sequencing read length distribution

Read-length distributions differed between the custom-primer libraries and datasets generated using the standard V1–V9 amplification protocol. In the Serteperti dog dataset produced with conventional ONT V1–V9 primers, read lengths formed a single peak centered around the expected full-length amplicon size (~1.5–1.6 kb), with no substantial secondary population (Supplementary Fig. S3). In contrast, the custom-primer libraries displayed a bimodal distribution consisting of the expected full-length peak and an additional population of shorter fragments (Supplementary Fig. S4).

Unlike the standard ONT 16S protocol, in which barcodes are incorporated into amplification primers, the custom workflow applied end repair followed by native barcode ligation after PCR. Under this strategy, any DNA fragment remaining after PCR cleanup will be ligated to a barcode and sequenced. The additional short-fragment peak therefore reflects the presence of non-target or truncated DNA molecules retained in the library.

## Supplementary information

Supplementary Figures and Tables

## Data Availability

All the raw sequencing data in fastq format and associated metadata were deposited to the European Nucleotide Archive (ENA) associated with the accessions • PRJEB75753^28^ (https://identifiers.org/ena.embl:PRJEB75753) for data obtained from Dog_M0 sample sequencing, • PRJEB82097^29^ (https://identifiers.org/ena.embl:PRJEB82097) for data from Serteperti cohort, • PRJEB85714^30^ (https://identifiers.org/ena.embl:PRJEB85714), and PRJEB85420^31^ (https://identifiers.org/ena.embl:PRJEB85420) for data generated from a human sample and synthetic and canine samples, respectively.

## Code availability

All scripts used for data analysis and visualization are publicly available in the GitHub repository: https://github.com/Balays/CaniMeta. The included R Markdown files document the workflow used to generate the reported analyses and visualizations.
