# Supplementary material for: Canine Fecal Microbiome Dataset: Ultra-deep Multi-platform Sequencing Across Extraction and Library Protocols
Source: Sci Data. 2026 Jun 9;13:1324. doi: 10.1038/s41597-026-07594-5 (PMC13586368; doi:10.1038/s41597-026-07594-5)
Supplement: Supplementary file 1 [file 41597_2026_7594_MOESM1_ESM.pdf]

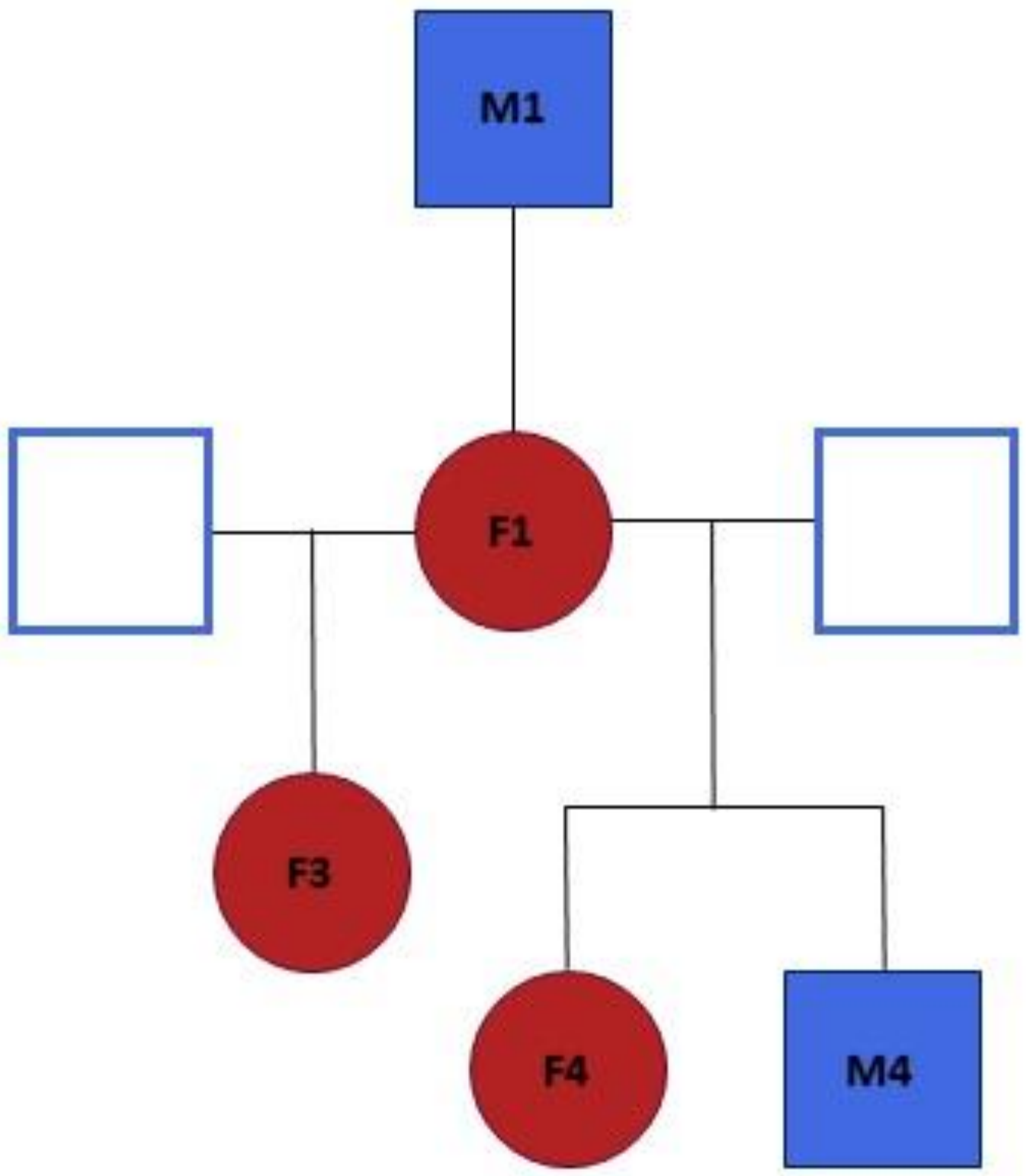

Supplementary Figure S1. Familial relationships among the dogs from the Serteperti cohort.

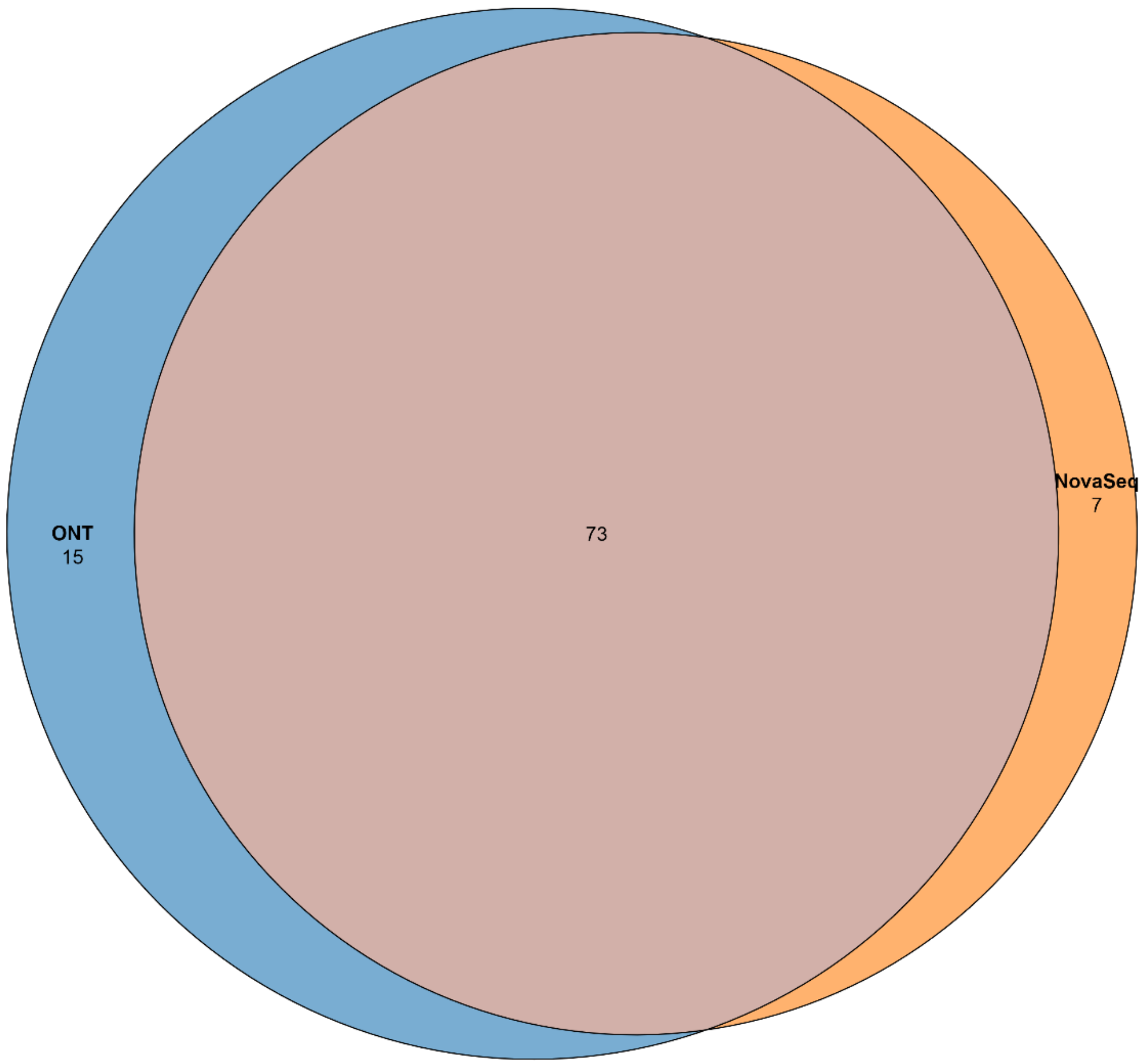

Supplementary Figure S2. Taxonomic overlap between sequencing platforms for the Dog_M0 sample.

Euler diagram showing the overlap of phyla detected in Oxford Nanopore MinION and Illumina NovaSeq datasets based on sourmash classification.

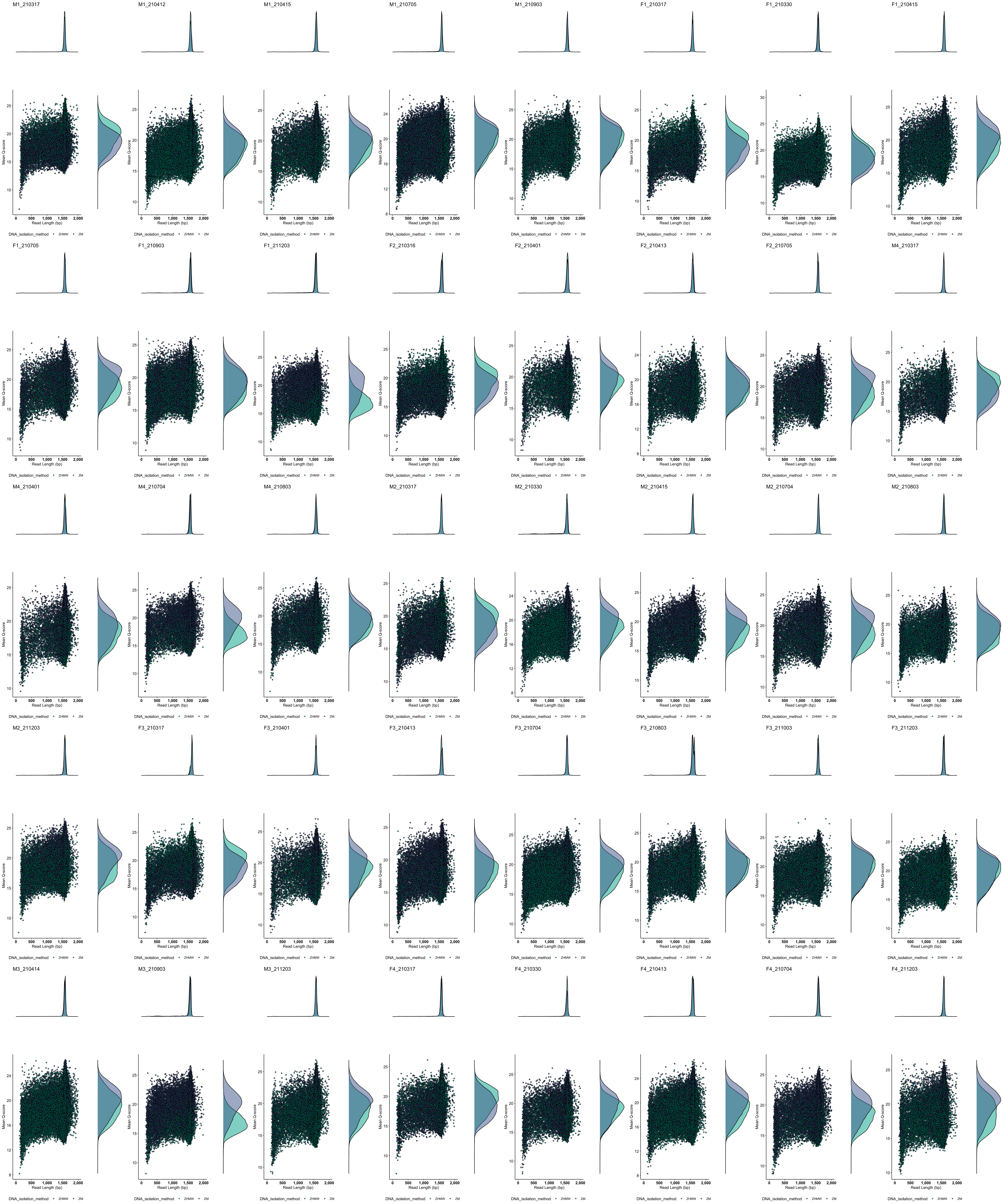
M1_210317
M1_210412
M1_210415
M1_210705
M1_210903
F1_210317
F1_210330
F1_210415
F1_210705
F1_210903
F1_211203
F2_210316
F2_210401
F2_210413
F2_210705
M4_210317
M4_210401
M4_210704
M4_210803
M2_210317
M2_210330
M2_210415
M2_210704
M2_210803
M2_211203
F3_210317
F3_210401
F3_210413
F3_210704
F3_210803
F3_211003
F3_211203
M3_210414
M3_210903
M3_211203
F4_210317
F4_210330
F4_210413
F4_210704
F4_211203
Mean Q-score
Read Length (bp)
0 500 1,000 1,500 2,000
DNA_isolation_method
ZHMW
ZM

Supplementary Figure S3. Read−length and quality characteristics of standard V1−V9 libraries

Read−level quality control metrics for libraries generated using the standard ONT full−length 16S rRNA gene amplification protocol (V1−V9 primers with integrated barcodes). Panels show read length distributions and the relationship between read length and mean per−read quality score across samples. Distributions are unimodal, with a dominant peak corresponding to the expected full−length amplicon (~1.5−1.6 kb), indicating successful enrichment of target molecules and minimal presence of off−target fragments.

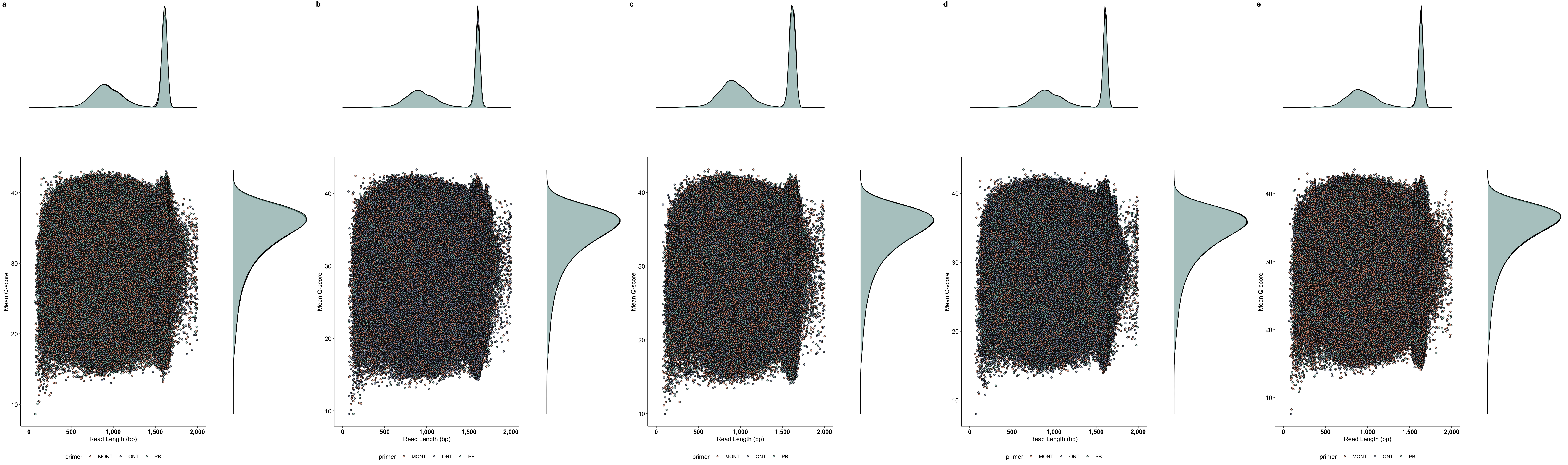
a
b
c
d
e
Mean Q-score
Read Length (bp)
primer MONT ONT PB

Supplementary Figure S4. Read−length and quality characteristics of custom−primer libraries

Quality control analysis of libraries generated using custom amplification primers followed by post−PCR end repair and native barcode ligation. Unlike the standard protocol, these libraries exhibit a bimodal read−length distribution comprising:

− a primary peak corresponding to full−length V1−V9 amplicons (~1.5−1.6 kb), and

− a secondary population of shorter fragments.

Scatter plots of read length versus mean quality score demonstrate that both fragment populations were sequenced with comparable quality characteristics. Density plots illustrate the mixed library composition at both per−sample and aggregate levels.

Panels are ordered by sample source as follows: (a) canine feces, (b) fecal reference material (FR), (c) gut microbiome standard (GMS), (d) microbial community standard (MCS), and (e) human feces. Each point represents an individual read. Marginal density plots depict the distributions of read length and quality within each panel. In all sample types, the dominant peak near ~1.5−1.6 kb corresponds to full−length V1−V9 16S rRNA gene amplicons.

Panel	Dog No.	Sex	Date of birth	Age (Years)	Sampling Date	Pregnancy related status	Litter birth date	Living place at sampling time
A	M0	Male	April 30, 2008	13.5	April 25, 2022	NA	NA	Szeged, Hungary
				14.8	August 12, 2023			
B	M1	Male	June 10, 2012	8.8	March 17, 2021			Dány, Hungary
				8.8	April 12, 2021			
				8.8	April 15, 2021			
				9.1	July 5, 2021			
				9.2	September 3, 2021			
	F1	Female	January 20, 2016	5.2	March 17, 2021			
				5.2	March 30, 2021			
				5.2	April 15, 2021			
				5.5	July 5, 2021			
				5.6	September 3, 2021			
				5.9	December 3, 2021			
	M2	Male	May 25, 2016	4.8	March 17, 2021			
				4.8	March 30, 2021			
				4.9	April 15, 2021			
				5.1	July 4, 2021			
				5.2	August 3, 2021			
				5.5	December 3, 2021			
	F2	Female	July 19, 2017	3.7	March 16, 2021			
				3.7	April 1, 2021			
				3.7	April 13, 2021			
				4.0	July 5, 2021	Pregnant		
				-	-	Whelping	August 3, 2021	
	F3	Female	December 30, 2017	-	-	Whelping	March 2, 2021	
				3.2	March 17, 2021	Lactation	NA	
				3.3	April 1, 2021	Lactation		
				3.3	April 13, 2021	Lactation		
				3.5	July 4, 2021	NA		
				3.6	August 3, 2021			
				3.8	October 3, 2021			
				3.9	December 3, 2021			
	M3	Male	August 8, 2018	2.7	April 14, 2021			
				3.1	September 3, 2021			
				3.3	December 3, 2021			
	F4	Female	February 1, 2019	2.1	March 17, 2021			
				2.2	March 30, 2021			
				2.2	April 13, 2021			
				2.4	July 4, 2021			
				2.8	December 3, 2021			
	M4	Male	February 1, 2019	2.1	March 17, 2021			
				2.2	April 1, 2021			
				2.4	July 4, 2021			
				2.5	August 3, 2021			
C	F5	Female	June 19, 2020	2.8	March 25, 2023			Ajka-Padragkút, Hungary

Supplementary Table S1.

Overview of the Pumi dogs included in the study, detailing their age, sampling dates, reproductive status, and living conditions at the time of sampling.

Instrument platform	Number of reads	Total bases	Mean read length	Median read length	N50 read length	mean read Q	median read Q	max read Q	min read Q
ILLUMINA	35 911 076	5 408 512 532	150.6	151	151	35.9	36.6	37.0	7.8
OXFORD_NANOPORE	2 956 194	3 784 325 241	1280.1	597	2599	31.4	32.3	44.6	4.8

Supplementary Table S2.

Read counts and other statistics on the Illumina NovaSeq and ONT MinION WGS sequencing data from the single-canine (Dog_M0) sample.

sample	biosample	DNA isolation method	project	Sample accession	Secondary sample accession	Sex	Date	Sample name	Dog	Read count
SRTP_A_M1_210317_ZHMW	ERR13927413	ZHMW	PRJEB82097	SAMEA116348872	ERS21409549	M	210317	M1_210317	M1	60579
SRTP_A_M1_210317_ZMB	ERR13927414	ZMB	PRJEB82097	SAMEA116348873	ERS21409550	M	210317	M1_210317	M1	103129
SRTP_A_M1_210412_ZHMW	ERR13927415	ZHMW	PRJEB82097	SAMEA116348874	ERS21409551	M	210412	M1_210412	M1	175244
SRTP_A_M1_210412_ZMB	ERR13927416	ZMB	PRJEB82097	SAMEA116348875	ERS21409552	M	210412	M1_210412	M1	51410
SRTP_A_M1_210415_ZHMW	ERR13927418	ZHMW	PRJEB82097	SAMEA116348876	ERS21409553	M	210415	M1_210415	M1	77937
SRTP_A_M1_210415_ZMB	ERR13927419	ZMB	PRJEB82097	SAMEA116348877	ERS21409554	M	210415	M1_210415	M1	62302
SRTP_A_M1_210705_ZHMW	ERR13927420	ZHMW	PRJEB82097	SAMEA116348878	ERS21409555	M	210705	M1_210705	M1	92530
SRTP_A_M1_210705_ZMB	ERR13927421	ZMB	PRJEB82097	SAMEA116348879	ERS21409556	M	210705	M1_210705	M1	136628
SRTP_A_M1_210903_ZHMW	ERR13927423	ZHMW	PRJEB82097	SAMEA116348881	ERS21409558	M	210903	M1_210903	M1	116742
SRTP_A_M1_210903_ZMB	ERR13927424	ZMB	PRJEB82097	SAMEA116348882	ERS21409559	M	210903	M1_210903	M1	62622
SRTP_A_F1_210317_ZHMW	ERR13926669	ZHMW	PRJEB82097	SAMEA116348888	ERS21409565	F	210317	F1_210317	F1	67692
SRTP_A_F1_210317_ZMB	ERR13926672	ZMB	PRJEB82097	SAMEA116348889	ERS21409566	F	210317	F1_210317	F1	97297
SRTP_A_F1_210330_ZHMW	ERR13926673	ZHMW	PRJEB82097	SAMEA116348890	ERS21409567	F	210330	F1_210330	F1	123820
SRTP_A_F1_210330_ZMB	ERR13926674	ZMB	PRJEB82097	SAMEA116348891	ERS21409568	F	210330	F1_210330	F1	53261
SRTP_A_F1_210415_ZHMW	ERR13926677	ZHMW	PRJEB82097	SAMEA116348892	ERS21409569	F	210415	F1_210415	F1	103053
SRTP_A_F1_210415_ZMB	ERR13926678	ZMB	PRJEB82097	SAMEA116348893	ERS21409570	F	210415	F1_210415	F1	66759
SRTP_A_F1_210705_ZHMW	ERR13926681	ZHMW	PRJEB82097	SAMEA116348895	ERS21409572	F	210705	F1_210705	F1	40346
SRTP_A_F1_210705_ZMB	ERR13926682	ZMB	PRJEB82097	SAMEA116348896	ERS21409573	F	210705	F1_210705	F1	99080
SRTP_A_F1_210903_ZHMW	ERR13926688	ZHMW	PRJEB82097	SAMEA116348899	ERS21409576	F	210903	F1_210903	F1	70212
SRTP_A_F1_210903_ZMB	ERR13926690	ZMB	PRJEB82097	SAMEA116348900	ERS21409577	F	210903	F1_210903	F1	64480
SRTP_A_F1_211203_ZHMW	ERR13926705	ZHMW	PRJEB82097	SAMEA116348904	ERS21409581	F	211203	F1_211203	F1	31756
SRTP_A_F1_211203_ZMB	ERR13926708	ZMB	PRJEB82097	SAMEA116348905	ERS21409582	F	211203	F1_211203	F1	55190
SRTP_A_F2_210316_ZHMW	ERR13927192	ZHMW	PRJEB82097	SAMEA116348907	ERS21409584	F	210316	F2_210316	F2	67907
SRTP_A_F2_210316_ZMB	ERR13927194	ZMB	PRJEB82097	SAMEA116348908	ERS21409585	F	210316	F2_210316	F2	107528
SRTP_A_F2_210401_ZHMW	ERR13927195	ZHMW	PRJEB82097	SAMEA116348909	ERS21409586	F	210401	F2_210401	F2	37067
SRTP_A_F2_210401_ZMB	ERR13927196	ZMB	PRJEB82097	SAMEA116348910	ERS21409587	F	210401	F2_210401	F2	40535
SRTP_A_F2_210413_ZHMW	ERR13927197	ZHMW	PRJEB82097	SAMEA116348911	ERS21409588	F	210413	F2_210413	F2	65379
SRTP_A_F2_210413_ZMB	ERR13927199	ZMB	PRJEB82097	SAMEA116348912	ERS21409589	F	210413	F2_210413	F2	50853
SRTP_A_F2_210705_ZHMW	ERR13927209	ZHMW	PRJEB82097	SAMEA116348915	ERS21409592	F	210705	F2_210705	F2	42808
SRTP_A_F2_210705_ZMB	ERR13927211	ZMB	PRJEB82097	SAMEA116348916	ERS21409593	F	210705	F2_210705	F2	95265
SRTP_A_M4_210317_ZHMW	ERR13927572	ZHMW	PRJEB82097	SAMEA116348926	ERS21409603	M	210317	M4_210317	M4	32503
SRTP_A_M4_210317_ZMB	ERR13927573	ZMB	PRJEB82097	SAMEA116348927	ERS21409604	M	210317	M4_210317	M4	50560
SRTP_A_M4_210401_ZHMW	ERR13927574	ZHMW	PRJEB82097	SAMEA116348928	ERS21409605	M	210401	M4_210401	M4	28281
SRTP_A_M4_210401_ZMB	ERR13927575	ZMB	PRJEB82097	SAMEA116348929	ERS21409606	M	210401	M4_210401	M4	51099
SRTP_A_M4_210704_ZHMW	ERR13927576	ZHMW	PRJEB82097	SAMEA116348930	ERS21409607	M	210704	M4_210704	M4	24370
SRTP_A_M4_210704_ZMB	ERR13927577	ZMB	PRJEB82097	SAMEA116348931	ERS21409608	M	210704	M4_210704	M4	118846
SRTP_A_M4_210803_ZHMW	ERR13927579	ZHMW	PRJEB82097	SAMEA116348932	ERS21409609	M	210803	M4_210803	M4	49550
SRTP_A_M4_210803_ZMB	ERR13927580	ZMB	PRJEB82097	SAMEA116348933	ERS21409610	M	210803	M4_210803	M4	61809
SRTP_A_M2_210317_ZHMW	ERR13927437	ZHMW	PRJEB82097	SAMEA116348942	ERS21409619	M	210317	M2_210317	M2	33960
SRTP_A_M2_210317_ZMB	ERR13927439	ZMB	PRJEB82097	SAMEA116348943	ERS21409620	M	210317	M2_210317	M2	52511
SRTP_A_M2_210330_ZHMW	ERR13927441	ZHMW	PRJEB82097	SAMEA116348944	ERS21409621	M	210330	M2_210330	M2	46080
SRTP_A_M2_210330_ZMB	ERR13927442	ZMB	PRJEB82097	SAMEA116348945	ERS21409622	M	210330	M2_210330	M2	40358
SRTP_A_M2_210415_ZHMW	ERR13927443	ZHMW	PRJEB82097	SAMEA116348946	ERS21409623	M	210415	M2_210415	M2	37630
SRTP_A_M2_210415_ZMB	ERR13927444	ZMB	PRJEB82097	SAMEA116348947	ERS21409624	M	210415	M2_210415	M2	120688
SRTP_A_M2_210704_ZHMW	ERR13927445	ZHMW	PRJEB82097	SAMEA116348948	ERS21409625	M	210704	M2_210704	M2	40675
SRTP_A_M2_210704_ZMB	ERR13927447	ZMB	PRJEB82097	SAMEA116348949	ERS21409626	M	210704	M2_210704	M2	100863
SRTP_A_M2_210803_ZHMW	ERR13927466	ZHMW	PRJEB82097	SAMEA116348951	ERS21409628	M	210803	M2_210803	M2	55023
SRTP_A_M2_210803_ZMB	ERR13927467	ZMB	PRJEB82097	SAMEA116348952	ERS21409629	M	210803	M2_210803	M2	57102
SRTP_A_M2_211203_ZHMW	ERR13927494	ZHMW	PRJEB82097	SAMEA116348958	ERS21409635	M	211203	M2_211203	M2	67395
SRTP_A_M2_211203_ZMB	ERR13927495	ZMB	PRJEB82097	SAMEA116348959	ERS21409636	M	211203	M2_211203	M2	50053
SRTP_A_F3_210317_ZHMW	ERR13927298	ZHMW	PRJEB82097	SAMEA116348964	ERS21409641	F	210317	F3_210317	F3	61883
SRTP_A_F3_210317_ZMB	ERR13927299	ZMB	PRJEB82097	SAMEA116348965	ERS21409642	F	210317	F3_210317	F3	94926
SRTP_A_F3_210401_ZHMW	ERR13927335	ZHMW	PRJEB82097	SAMEA116348968	ERS21409645	F	210401	F3_210401	F3	40409
SRTP_A_F3_210401_ZMB	ERR13927337	ZMB	PRJEB82097	SAMEA116348969	ERS21409646	F	210401	F3_210401	F3	42105
SRTP_A_F3_210413_ZHMW	ERR13927339	ZHMW	PRJEB82097	SAMEA116348970	ERS21409647	F	210413	F3_210413	F3	32070
SRTP_A_F3_210413_ZMB	ERR13927340	ZMB	PRJEB82097	SAMEA116348971	ERS21409648	F	210413	F3_210413	F3	66769
SRTP_A_F3_210704_ZHMW	ERR13927346	ZHMW	PRJEB82097	SAMEA116348973	ERS21409650	F	210704	F3_210704	F3	132552
SRTP_A_F3_210704_ZMB	ERR13927348	ZMB	PRJEB82097	SAMEA116348974	ERS21409651	F	210704	F3_210704	F3	116984
SRTP_A_F3_210803_ZHMW	ERR13927355	ZHMW	PRJEB82097	SAMEA116348976	ERS21409653	F	210803	F3_210803	F3	111529
SRTP_A_F3_210803_ZMB	ERR13927357	ZMB	PRJEB82097	SAMEA116348977	ERS21409654	F	210803	F3_210803	F3	63691
SRTP_A_F3_211003_ZHMW	ERR13927361	ZHMW	PRJEB82097	SAMEA116348978	ERS21409655	F	211003	F3_211003	F3	112393
SRTP_A_F3_211003_ZMB	ERR13927362	ZMB	PRJEB82097	SAMEA116348979	ERS21409656	F	211003	F3_211003	F3	60656
SRTP_A_F3_211203_ZHMW	ERR13927370	ZHMW	PRJEB82097	SAMEA116348982	ERS21409659	F	211203	F3_211203	F3	136981
SRTP_A_F3_211203_ZMB	ERR13927371	ZMB	PRJEB82097	SAMEA116348983	ERS21409660	F	211203	F3_211203	F3	56908
SRTP_A_M3_210414_ZHMW	ERR13927502	ZHMW	PRJEB82097	SAMEA116348987	ERS21409664	M	210414	M3_210414	M3	156597
SRTP_A_M3_210414_ZMB	ERR13927503	ZMB	PRJEB82097	SAMEA116348988	ERS21409665	M	210414	M3_210414	M3	56238
SRTP_A_M3_210903_ZHMW	ERR13927504	ZHMW	PRJEB82097	SAMEA116348989	ERS21409666	M	210903	M3_210903	M3	24404
SRTP_A_M3_210903_ZMB	ERR13927505	ZMB	PRJEB82097	SAMEA116348990	ERS21409667	M	210903	M3_210903	M3	50090
SRTP_A_M3_211203_ZHMW	ERR13927531	ZHMW	PRJEB82097	SAMEA116348993	ERS21409670	M	211203	M3_211203	M3	112462
SRTP_A_M3_211203_ZMB	ERR13927532	ZMB	PRJEB82097	SAMEA116348994	ERS21409671	M	211203	M3_211203	M3	47206
SRTP_A_F4_210317_ZHMW	ERR13927381	ZHMW	PRJEB82097	SAMEA116348997	ERS21409674	F	210317	F4_210317	F4	32963
SRTP_A_F4_210317_ZMB	ERR13927382	ZMB	PRJEB82097	SAMEA116348998	ERS21409675	F	210317	F4_210317	F4	41814
SRTP_A_F4_210330_ZHMW	ERR13927383	ZHMW	PRJEB82097	SAMEA116348999	ERS21409676	F	210330	F4_210330	F4	44552
SRTP_A_F4_210330_ZMB	ERR13927386	ZMB	PRJEB82097	SAMEA116349000	ERS21409677	F	210330	F4_210330	F4	43774
SRTP_A_F4_210413_ZHMW	ERR13927388	ZHMW	PRJEB82097	SAMEA116349001	ERS21409678	F	210413	F4_210413	F4	163624
SRTP_A_F4_210413_ZMB	ERR13927389	ZMB	PRJEB82097	SAMEA116349002	ERS21409679	F	210413	F4_210413	F4	63410
SRTP_A_F4_210704_ZHMW	ERR13927390	ZHMW	PRJEB82097	SAMEA116349003	ERS21409680	F	210704	F4_210704	F4	33949
SRTP_A_F4_210704_ZMB	ERR13927392	ZMB	PRJEB82097	SAMEA116349004	ERS21409681	F	210704	F4_210704	F4	121264
SRTP_A_F4_211203_ZHMW	ERR13927406	ZHMW	PRJEB82097	SAMEA116349010	ERS21409687	F	211203	F4_211203	F4	69401
SRTP_A_F4_211203_ZMB	ERR13927407	ZMB	PRJEB82097	SAMEA116349011	ERS21409688	F	211203	F4_211203	F4	51059

Supplementary Table S3.

Read counts and other information on the ZMB–ZHMW DNA isolation kit

Sample	primer	source	Read number	Total bases	Mean length	Median length	N50 length	Max length	Min length	Mean Q	Median Q	biosample	project	Sample accession	Secondary sample accession
PRMT_CO1_canis_ONT	ONT	canis	367 092	461 185 684	1256.3	1185	1586	56230	83	33.8	34.8	ERR14294149	PRJEB85420	SAMEA117664628	ERS23179863
PRMT_CO2_canis_ONT	ONT	canis	258 773	324 620 172	1254.5	1175	1586	24316	82	33.8	34.9	ERR14294152	PRJEB85420	SAMEA117664629	ERS23179864
PRMT_CO3_canis_ONT	ONT	canis	272 227	341 128 966	1253.1	1173	1582	27290	83	33.6	34.6	ERR14294156	PRJEB85420	SAMEA117664630	ERS23179865
PRMT_CP1_canis_PB	PB	canis	300 312	373 270 355	1242.9	1184	1587	109030	86	33.9	34.9	ERR14294158	PRJEB85420	SAMEA117664634	ERS23179869
PRMT_CP2_canis_PB	PB	canis	573 305	713 379 981	1244.3	1185	1587	67311	71	33.9	34.9	ERR14294175	PRJEB85420	SAMEA117664635	ERS23179870
PRMT_CP3_canis_PB	PB	canis	353 477	438 753 176	1241.2	1180	1585	89660	82	33.8	34.8	ERR14294180	PRJEB85420	SAMEA117664636	ERS23179871
PRMT_FM1_FR_MONT	MONT	FR	352 992	439 999 929	1246.5	1194	1594	57117	92	33.8	34.9	ERR14250963	PRJEB85420	SAMEA117659009	ERS23137326
PRMT_FM2_FR_MONT	MONT	FR	452 286	566 005 228	1251.4	1214	1594	119262	78	33.9	34.9	ERR14250964	PRJEB85420	SAMEA117659010	ERS23137327
PRMT_FM3_FR_MONT	MONT	FR	289 757	361 718 753	1248.4	1202	1594	38621	81	33.8	34.8	ERR14250965	PRJEB85420	SAMEA117659011	ERS23137328
PRMT_FO1_FR_ONT	ONT	FR	523 840	657 685 086	1255.5	1207	1593	122031	86	33.7	34.7	ERR14250966	PRJEB85420	SAMEA117659000	ERS23137317
PRMT_FO2_FR_ONT	ONT	FR	700 037	883 251 669	1261.7	1214	1593	53634	71	33.8	34.9	ERR14250979	PRJEB85420	SAMEA117659001	ERS23137318
PRMT_FO3_FR_ONT	ONT	FR	755 429	949 024 627	1256.3	1211	1593	88140	79	33.7	34.8	ERR14250980	PRJEB85420	SAMEA117659002	ERS23137319
PRMT_FP1_FR_PB	PB	FR	218 212	271 685 170	1245.1	1191	1592	40080	75	33.8	34.9	ERR14250981	PRJEB85420	SAMEA117659018	ERS23137335
PRMT_FP2_FR_PB	PB	FR	210 590	261 601 086	1242.2	1182	1589	125032	85	33.8	34.8	ERR14250982	PRJEB85420	SAMEA117659019	ERS23137336
PRMT_FP3_FR_PB	PB	FR	219 522	273 401 901	1245.4	1191	1592	16198	86	33.8	34.9	ERR14250983	PRJEB85420	SAMEA117659020	ERS23137337
PRMT_GM1_GMS_MONT	MONT	GMS	455 374	571 034 237	1254.0	1191	1593	53712	78	34.0	35.0	ERR14250984	PRJEB85420	SAMEA117659006	ERS23137323
PRMT_GM2_GMS_MONT	MONT	GMS	458 325	575 087 288	1254.8	1207	1592	38344	88	33.8	34.8	ERR14250985	PRJEB85420	SAMEA117659007	ERS23137324
PRMT_GM3_GMS_MONT	MONT	GMS	58 120	70 580 155	1214.4	1107	1561	75274	109	33.9	34.9	ERR14250986	PRJEB85420	SAMEA117659008	ERS23137325
PRMT_GO1_GMS_ONT	ONT	GMS	424 379	534 525 845	1259.5	1186	1592	28106	78	33.9	34.9	ERR14250987	PRJEB85420	SAMEA117658997	ERS23137314
PRMT_GO2_GMS_ONT	ONT	GMS	442 719	557 330 002	1258.9	1195	1593	35257	85	33.7	34.7	ERR14250988	PRJEB85420	SAMEA117658998	ERS23137315
PRMT_GO3_GMS_ONT	ONT	GMS	191 637	241 838 063	1262.0	1163	1590	121951	81	33.8	34.8	ERR14250989	PRJEB85420	SAMEA117658999	ERS23137316
PRMT_GP1_GMS_PB	PB	GMS	271 918	338 570 723	1245.1	1170	1592	16237	96	33.9	34.9	ERR14250999	PRJEB85420	SAMEA117659015	ERS23137332
PRMT_GP2_GMS_PB	PB	GMS	189 653	235 714 377	1242.9	1171	1590	17842	84	33.8	34.8	ERR14251000	PRJEB85420	SAMEA117659016	ERS23137333
PRMT_GP3_GMS_PB	PB	GMS	288 406	360 591 839	1250.3	1190	1592	17022	87	33.8	34.8	ERR14251001	PRJEB85420	SAMEA117659017	ERS23137334
PRMT_HM1_human_MONT	MONT	human	415 725	518 822 406	1248.0	1194	1598	60419	63	33.7	34.7	ERR14365448	PRJEB85714	SAMEA117686893	ERS23237318
PRMT_HM2_human_MONT	MONT	human	285 588	356 348 911	1247.8	1197	1598	97189	98	33.4	34.4	ERR14365449	PRJEB85714	SAMEA117686894	ERS23237319
PRMT_HM3_human_MONT	MONT	human	265 339	330 464 238	1245.4	1186	1597	57727	77	33.4	34.4	ERR14365450	PRJEB85714	SAMEA117686895	ERS23237320
PRMT_HO1_human_ONT	ONT	human	868 183	1 085 944 708	1250.8	1204	1597	64893	76	33.6	34.6	ERR14365451	PRJEB85714	SAMEA117686890	ERS23237315
PRMT_HO2_human_ONT	ONT	human	329 316	415 204 241	1260.8	1178	1594	30327	74	33.4	34.3	ERR14365452	PRJEB85714	SAMEA117686891	ERS23237316
PRMT_HO3_human_ONT	ONT	human	472 784	592 612 176	1253.5	1193	1596	71426	76	33.7	34.7	ERR14365453	PRJEB85714	SAMEA117686892	ERS23237317
PRMT_HP1_human_PB	PB	human	287 609	357 991 006	1244.7	1186	1598	27389	67	33.7	34.7	ERR14365459	PRJEB85714	SAMEA117686896	ERS23237321
PRMT_HP2_human_PB	PB	human	171 610	213 505 468	1244.1	1186	1596	17698	80	33.4	34.4	ERR14365470	PRJEB85714	SAMEA117686897	ERS23237322
PRMT_HP3_human_PB	PB	human	439 960	548 002 371	1245.6	1190	1597	38603	85	33.6	34.6	ERR14365508	PRJEB85714	SAMEA117686898	ERS23237323
PRMT_LM1_canis_MONT	MONT	canis	246 077	305 900 417	1243.1	1169	1584	24032	88	33.9	34.9	ERR14294189	PRJEB85420	SAMEA117664631	ERS23179866
PRMT_LM2_canis_MONT	MONT	canis	673 209	838 861 696	1246.1	1189	1588	40180	84	33.7	34.7	ERR14294191	PRJEB85420	SAMEA117664632	ERS23179867
PRMT_LM3_canis_MONT	MONT	canis	595 099	741 270 380	1245.6	1188	1587	37355	81	33.9	34.9	ERR14294201	PRJEB85420	SAMEA117664633	ERS23179868
PRMT_MM1_MCS_MONT	MONT	MCS	567 673	713 997 528	1257.8	1188	1625	57070	90	34.1	35.1	ERR14251002	PRJEB85420	SAMEA117659003	ERS23137320
PRMT_MM2_MCS_MONT	MONT	MCS	339 721	429 960 361	1265.6	1189	1625	24865	79	34.2	35.2	ERR14251003	PRJEB85420	SAMEA117659004	ERS23137321
PRMT_MM3_MCS_MONT	MONT	MCS	508 327	641 369 670	1261.7	1195	1625	138764	74	34.2	35.2	ERR14251004	PRJEB85420	SAMEA117659005	ERS23137322
PRMT_MO1_MCS_ONT	ONT	MCS	323 460	408 251 753	1262.1	1181	1622	150326	68	34.1	35.2	ERR14251005	PRJEB85420	SAMEA117658994	ERS23137311
PRMT_MO2_MCS_ONT	ONT	MCS	322 123	405 950 673	1260.2	1181	1622	13574	81	34.2	35.3	ERR14251007	PRJEB85420	SAMEA117658995	ERS23137312
PRMT_MO3_MCS_ONT	ONT	MCS	432 025	543 023 579	1256.9	1183	1621	53417	78	34.0	35.0	ERR14251008	PRJEB85420	SAMEA117658996	ERS23137313
PRMT_MP1_MCS_PB	PB	MCS	230 510	289 060 511	1254.0	1184	1623	22115	92	34.2	35.3	ERR14251009	PRJEB85420	SAMEA117659012	ERS23137329
PRMT_MP2_MCS_PB	PB	MCS	333 648	418 421 393	1254.1	1183	1624	59552	83	34.2	35.2	ERR14251019	PRJEB85420	SAMEA117659013	ERS23137330
PRMT_MP3_MCS_PB	PB	MCS	210 546	262 983 050	1249.1	1163	1623	15606	80	34.1	35.1	ERR14251020	PRJEB85420	SAMEA117659014	ERS23137331

Supplementary Table S4.

Read counts and other statistics on the primer comparison dataset.
